# Overview of Liquid Crystal Biosensors: From Basic Theory to Advanced Applications

**DOI:** 10.3390/bios12040205

**Published:** 2022-03-29

**Authors:** Ruixiang Qu, Guoqiang Li

**Affiliations:** Intelligent Optical Imaging and Sensing Group, Zhejiang Laboratory, Hangzhou 311121, China; qrx21@zhejianglab.edu.cn

**Keywords:** liquid crystal, biosensor, optical sensing, active optical components

## Abstract

Liquid crystals (LCs), as the remarkable optical materials possessing stimuli-responsive property and optical modulation property simultaneously, have been utilized to fabricate a wide variety of optical devices. Integrating the LCs and receptors together, LC biosensors aimed at detecting various biomolecules have been extensively explored. Compared with the traditional biosensing technologies, the LC biosensors are simple, visualized, and efficient. Owning to the irreplaceable superiorities, the research enthusiasm for the LC biosensors is rapidly rising. As a result, it is necessary to overview the development of the LC biosensors to guide future work. This article reviews the basic theory and advanced applications of LC biosensors. We first discuss different mesophases and geometries employed to fabricate LC biosensors, after which we introduce various detecting mechanisms involved in biomolecular detection. We then focus on diverse detection targets such as proteins, enzymes, nucleic acids, glucose, cholesterol, bile acids, and lipopolysaccharides. For each of these targets, the development history and state-of-the-art work are exhibited in detail. Finally, the current challenges and potential development directions of the LC biosensors are introduced briefly.

## 1. Introduction

Liquid crystals (LCs) are remarkable optical materials that exist in the case of certain substances located between solid and liquid states, typically composed of elongated organic molecules with anisotropic shapes [1,2,3,4,5]. They possess liquid-like fluidity and solid-like ordering simultaneously and have aroused wide interest due to their fascinating performances. Various mesophases of LCs such as the nematic LCs [6,7], chiral nematic LCs [8,9], blue phase LCs [10,11], and ionic LCs [12,13] have been explored and investigated in-depth since the first LC was observed by F. Reinitzer in 1888 [14,15]. The main driving force of the orderly arrangement has been proven to be the elastic force between LC molecules, which is relatively weak and makes it feasible to change the arrangement by different external stimuli [16,17]. As a result, the LCs show an intrinsic responsive property that can be triggered by the magnetic field [18], electric field [19], and external molecules [20], promising them outstanding potential in fabricating responsive materials and devices. Additionally, due to their anisotropic shapes, LCs are found to be optically anisotropic, enabling them to realize the optical modulation by diverse approaches including birefringence [21,22], scattering [23,24], and reflection [25,26]. Integrating the optical modulation property and the stimuli-responsive property together, the applications of LCs in a wide variety of technological applications such as beam splitters [27,28], lens [29,30], displays [31,32], color filters [33,34], gratings [35,36], and actuators [37] have been proposed. Worth mentioning is that the LCs are classified into two types including the thermotropic LCs and lyotropic LCs, based on the phase transition condition [38]. In this review, we are interested in the thermotropic LCs because of their application potential in biosensing.

Over the past several years, LC sensors have become feasible and widely used tools to detect analytes such as biomolecules [39,40], gases [41,42], ions [43,44], light [45], and temperature [46,47,48], among which the detection of biomolecules show extraordinary attraction in disease diagnosis, health monitoring, and cell simulation. The working principle of the LC biosensors mainly relies on the synergistic effect of the LC molecules and subtly chosen receptors. The receptors, which play the role of the transmitters, can combine with the biomolecules and pass the molecule signals to LCs. The LCs acting as the processors can transform the biomolecule signals into optical signals via optical modulation. Traditional biosensing technologies such as the metal–oxide–semiconductor sensors [49,50], electrochemical sensors [51,52], and light addressable potentiometric sensors [53,54] usually call for precise instruments, professional operators, and low detection flux. In comparison, the LC biosensors provide an alternative approach to realizing the simple and efficient biomolecular detection, as well as a visualized output signal that can be directly observed by the naked eye using some optical techniques. Owning to the irreplaceable superiorities, LC biosensors have been reported to detect a wide range of biomolecules. As a result, it is necessary to summarize the current work and outlook the development directions of the LC biosensors, laying the foundation for future research. Although the early research progress of the LC biosensors has been reviewed by the Abbott group in 2013 [55], a cutting-edge review is still necessary to overview the new developments in methods and advanced applications of this field. On the other hand, the design and optimization of the LC biosensors have also been reviewed by the Abbott group and the Jakli group [56,57], and those contents are outside the scope of this work.

**Figure 1:**
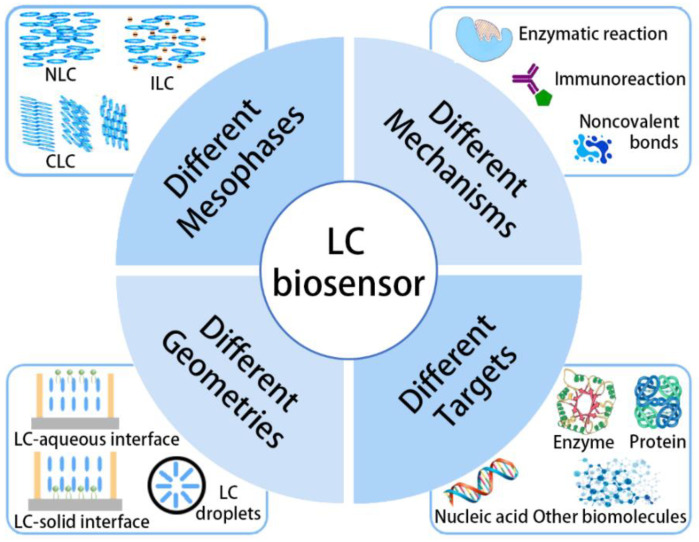
Schematic showing the overall outline of this review, including the mesophases, mechanisms, geometries and detection targets of the LC biosensor.

To take advantage of the LC biosensors, various mesophases have been utilized to fabricate the sensors. The geometries of the biosensors, which can impose the specific LC alignments and influence the detection property, are also studied. The intrinsic detection mechanisms, based on diverse intermolecular interactions, have been investigated in depth. In this review, we first summarize the basic knowledge about the sensor structures and detection mechanisms, aimed at establishing basic concepts of the LC biosensors. Some common mesophases, including the nematic liquid crystals (NLCs), chiral nematic liquid crystals (CLCs), and ionic liquid crystals (ILCs), accompanied by three different geometries, are exhibited in Section 2. Various intermolecular interactions involved in the biomolecular detections, such as the enzymatic reactions, immunoreactions, and noncovalent interactions, are discussed in Section 3. We then focus on the current hot detection targets of the LC biosensors including proteins, enzymes, nucleic acids, glucose, cholesterol, bile acids, and lipopolysaccharides in Section 4. For each of the targets, the development history and research status of the biosensors are exhibited in detail. Furthermore, the current challenges and potential development directions of the LC biosensors are introduced briefly in Section 5, which can provide guidance for future research. The overall outline of this review is shown in Figure 1.

## 2. Different Sensor Structures

The properties of the LC biosensors, especially the limit of detection (LOD), detection rate, and optical signal, are strongly related to the sensor structure. Up to now, mesophases such as NLCs and CLCs have been widely used to fabricate the LC biosensors, among which the former exhibit diverse optical signals under the polarizing microscope (POM) via birefringence [58], and the latter possess tunable reflection color that can be directly observed by naked eyes [59]. Other mesophases such as the smectic phase and blue phase are rarely employed in designing biosensors, although their application in the field of LC display, LC lens, LC gratings, and LC color filters have been proven to be promising [60,61]. In recent years, with the development of ionic liquids, ILCs, born at the interface of ionic liquids and liquid crystals, have aroused great attention. Some LC biosensors based on the ILCs have been proposed, holding satisfactory electrical properties that can be tuned by biological signals. The subtle choice of mesophases can help to obtain the LC biosensors with presupposed performances. Additionally, the role of carefully choosing the geometry of the sensors is to impose a specific alignment, which can propagate over the macroscopic distances within the LC phase, and confine the LC in a designated space. Three different geometries including the LC–aqueous interface [62,63], the LC–solid interface [64,65], and the LC droplets with spherical interface [66,67] have been investigated deeply. In this section, we will discuss different mesophases and geometries used to fabricate LC biosensors, aimed at establishing the basic concepts of designing the devices.

### 2.1. Mesophases

#### 2.1.1. Nematic LCs

NLCs, as the simplest and most common mesophase of LCs, are typically formed by elongated rodlike molecules which possess parallel orientation along with the director. The director is an axis of full rotational symmetry, making the NLCs nonpolar and uniaxial. The molecules do not have long-range order that they can slide along multiple directions. The short-range interaction among the NLC molecules has been proven to be the Van der Waals force, which is weak and allows the high fluidity of the molecules [68]. For an actual LC phase, the LC molecules are not ideally parallel, showing fluctuation around the director. The order parameter *S*, which is used to quantify the degree of molecular orientation relative to the director, have been proposed and defined by the equation below,

(1) $$S=\frac{1}{2}\langle 3\cos^{2}\alpha -1\rangle .$$

In this equation, *α* means the angle between the long molecular axis and the director, and the brackets denote the average over all molecules [69]. For a typical crystal with ideal ordering, the order parameter is 1. While for an isotropic fluid, in which the molecules order randomly, the order parameter is 0. For the NLCs, the order parameter usually ranges from 0.5 to 0.7. Since the NLCs hold high ordering as well as weak intermolecular force, the tuning of the NLC alignment is feasible, which lays the foundation for fabricating NLC biosensors.

#### 2.1.2. Chiral Nematic LCs

By doping an NLC with chiral molecules, another mesophase that has been widely employed in biosensing, known as the CLC, can be obtained. The chiral molecules are the molecules possessing chiral centers and have a steric configuration that cannot be superimposed on its mirror image [70]. Typically speaking, the molecules in a CLC have a director that is periodically rotated along the perpendicular axis, forming a helical superstructure with either a left-handed or right-handed configuration [71]. Such helical superstructures provide the CLCs with the periodic modulation of refractive index along the helical axis, therefore leading to the photonic bandgap (PBG) [72]. The central wavelength of the PBG depends on the incident angle and the intrinsic properties of the CLC, and can be estimated by Bragg’s law,

(2) λ=nP cos β,

where *n* is the average refractive index, *P* is the helical pitch, and *β* is the incident angle [73]. At normal incidence, the bandwidth of the reflected light is related to the refractive index and the pitch, and can be calculated by the equation below,

(3) $$\Delta \lambda =P\left(n_{e}-n_{o}\right),\;$$

where *n_o_* and *n_e_* are the ordinary and extraordinary refractive indices of the LCs [74]. By regulating the amount of the chiral molecules, the bandwidth and the central wavelength can be easily tuned, greatly enhancing the versatility of the CLCs. Based on the CLCs, the LC biosensors that can directly express color signals have been designed, promising the POM-free detection of the biomolecules.

#### 2.1.3. Smectic LCs and Blue Phase LCs

Smectic LC is also a common mesophase that has been extensively explored, possessing a layered structure with well-defined periodicity. Among all the mesophases, smectic LCs have the highest ordering and strongest intermolecular forces. To measure the ordering of the smectic LCs, the parameter named smectic order parameter is defined by the equation below,

(4) $$\sigma =\langle \frac{3\cos^{2}\alpha -1}{2}\cos\left(\frac{2\pi }{d}z\right)\rangle ,\;$$

where *α* is the angle between the director and the long molecular axis, *d* is the layer spacing, and *z* is the position of the molecule center [41]. Up to now, a variety of smectic LCs such as SmA, SmB, and SmC have been deeply investigated, while their applications in the field of biosensing are still difficult, which is because of their strong intermolecular forces [75,76,77]. Moreover, blue phase LCs are commonly observed between the isotropic phase and CLC phase in strong chiral systems, possessing cubic structures with double-twisted cylinders and showing PBGs that rely on the blue phase helixes [78]. However, they only exist within a narrow temperature range, which is severely hindering their development in biosensing [79].

#### 2.1.4. Ionic LCs

Over the past decade, the field of ILCs has rapidly developed, and efforts have been devoted to designing LC biosensors using the ILCs. Typically speaking, the ILCs are fabricated by introducing ionic groups into the LC molecules, forming the mesomorphic salts, and endowing LCs with ionic liquid-like properties. The driving forces for stabilizing ILCs have been proven to be the electrostatic interactions between cations and anions, as well as the micro-phase segregation of incompatible units [80]. Holding the orientation of LCs and the electrical conductivity of ions at the same time, the ILCs have been widely used in the field of ionic conductive materials, organic reaction agents, and functional nanomaterials. Different from the traditional LC optical sensors, for the ILC biosensors, the chemical signals of the biomolecules can be observed by measuring the electrical properties.

### 2.2. Geometries

The detection of biomolecules using the LC biosensors relies on tuning the LC alignment. The role of choosing the geometry of the sensors is to confine the LCs in a designated space with a specific interface, and therefore to impose a specific LC alignment. As a result, the geometries of the sensor can greatly influence the detection property. According to the binding site of the alignment layer, three kinds of different geometries have been investigated, including the LC–solid interface, the LC–aqueous interface, and the LC droplets with a spherical interface.

#### 2.2.1. LC–Solid Interface

For the LC biosensors based on the LC–solid interface, the alignment agents such as polyimide, azo derivatives, oligonucleotides, and metal nanoparticles are usually modified on the glass substrates by the covalent bonds [81,82], leading to the homeotropic or parallel alignment of the LCs, as well as giving rise to a range of intermolecular interactions between the interface and the detection targets. For a real LC–solid interface, the LC orientation at the interface is usually not ideally uniform, and parameters including zenithal (*θ*) and azimuthal (*ϕ*) angles are proposed to measure the LC director at the interface [83]. The zenithal angle means the angle between the z axis and the LC director, while the azimuthal angle signifies the angle between the x axis and the LC director. When *θ* = 0°, the LC director is perpendicular to the surface, corresponding to the perfect homeotropic alignment. When *θ* = 90°, the LC director is parallel to the surface with the LC molecules in the parallel alignment. The LC alignment at the LC–solid interface can directly impact the optical property of the LC phase, which can be measured by the optical retardation of the light, as well as the transmittance of the LC cell sandwiched between two orthogonal polarizers. The optical retardation can be calculated by the effective refractive index change described below,

(5) $$n_{\mathrm{eff}}=\frac{n_{o}n_{e}}{\sqrt{n_{e}^{2}\cos^{2}\theta +n_{o}^{2}\sin^{2}\theta }},\;$$

where *n*_eff_ is the effective refractive index, *n_e_* and *n_o_* are the extraordinary and ordinary refractive index of the LCs, and *θ* is the zenithal angle mentioned above. In general, the phase retardation *τ* can be expressed by the equation,

(6) $$\tau =\int_{0}^{l}\frac{2\pi n_{\mathrm{eff}}\left(z\right)}{\lambda }dz,\;$$

where *l* is the thickness of the LC cell and *λ* is the wavelength of light. Based on the phase retardation, the transmittance of the light can be given by

(7) $$T=\frac{I}{I_{o}}=\frac{I_{o}\sin^{2}\frac{\tau }{2}}{I_{o}},\;$$

in which *T* is the transmittance, *I* and *I_o_* are the intensity of the transmission light and the incident light, respectively [84]. As a result, in order to obtain an LC–solid interface with presupposed property, the device structure should be subtly designed.

#### 2.2.2. LC-Aqueous Interface

For the LC biosensors based on the LC–aqueous interface, the alignment agents and the receptors are usually immobilized at the interface via the noncovalent bonds such as the hydrophobic interaction, electrostatic interaction, and coordination interaction [85,86]. Amphiphilic surfactants have been proven to be the alternative candidate for the alignment agent, possessing the ability to trigger the homeotropic alignment of the LCs with their hydrophilic heads combined with water, and their hydrophobic tails combined with the LCs [87]. In the absence of the surfactants, however, the LC molecules align in parallel under the influence of the induced dipole–dipole interaction with the water phase. Up to now, there have been a variety of studies reporting the LC biosensors based on amphiphilic surfactants. For example, by utilizing the electrostatic interaction between the surfactant and the oligonucleotide, an LC nucleic acid sensor has been achieved [88]. Based on the protonation and deprotonation of the surfactant, a biosensor that can image enzymatic reaction is proposed [89]. For detecting specific protein–protein binding, a LC–aqueous interface decorated by a Ni-modified surfactant has been designed [90].

In order to pave the way for optimizing the LC biosensors based on the LC–aqueous interface, the Jakli group has studied in-depth the influences of the LC elastic constants and surface anchoring energy on the sensitivity of biosensors [91]. It is found that tuning the elastic constant by mixing different LC molecules can realize an almost linear relationship between the surfactant concentration and the effective birefringence. Reducing the anchoring strength by changing the alignment layer can increase the critical concentration of the surfactant, and therefore provide a wider sensing range. The relationship between the anchoring strength and the critical surfactant concentration can be described by the equation,

(8) $$c=c_{s}\left(1-\frac{b_{t}}{L}+\frac{b_{b}b_{t}}{L}\right)$$

where *L* is the thickness of the LC phase, *c_s_* is the saturated concentration of the surfactant, *b_t_* and *b_b_* are the extrapolation lengths of the top and bottom surfaces, respectively. The parameter *b_b_* is negatively correlated with the anchoring strength of the alignment layer. This study reveals the importance of tuning both the LCs and the alignment layer in fabricating the LC biosensors based on the LC–aqueous interface.

Compared with the LC–solid interface, the LC–aqueous interface can permit the biosensors with higher sensitivity that the detection target can directly contact with the receptor without the intercept of the LC phase. However, due to the absence of a solid surface, the LC–aqueous interface is not capable of achieving the sensing microarray that can be used to detect thousands of targets in a single experiment.

#### 2.2.3. LC Droplets

Aiming at improving the specific surface area and achieving a lower LOD, the LC droplets with a spherical interface have been proposed. The LC droplets are usually fabricated by emulsifying techniques such as stirring emulsification [92], membrane emulsification [93], and microfluidic encapsulation [94]. The stirring emulsification is the simplest strategy to prepare the LC droplets in bulk, while the obtained droplets hold broad size distribution. The size of the LC droplets can be tuned by adjusting the stirring rate and stirring time. Additionally, membrane emulsification can produce LC droplets with uniform size, which is dependent on the pore size of the membrane. Among all the strategies, microfluidic encapsulation can obtain the LC droplets with the most uniform size, while the yield remains to be improved.

The LCs encapsulated in the droplets are reorientated under the influence of the spherical geometry and thus can produce the radial configuration, bipolar configuration, or pre-radial configuration, dictated by the balance of the elastic and interfacial contributions to the free energy [95,96,97]. As the spherical geometry cannot sustain a continuous strain of the LCs, different topological defects are formed to reduce the free energy and stabilize the droplet. For the bipolar configuration, two point defects in the two poles of the droplets named boojums can be observed [98]. For the radial configuration, a central point defect called hedgehog will appear [99].

For the CLC droplets [100], the Frank–Pryce configuration and the nested cup configuration are the most common, among which the former signifies the planar alignment of the LC molecules, and the latter means the perpendicular alignment. Paterson et al. studied the configuration transition of the CLC droplets using various orientation agents including phospholipids, sodium dodecyl sulfate (SDS), and polyvinyl alcohol (PVA) [100]. It is found that PVA favors the Frank–Pryce configuration, while SDS favors the nested cup configuration. Further, different phospholipids can lead to different configurations of the CLC droplets. For example, the mixture of DOPC and DOPG can result in the nested cup configuration for most of the CLCs, and the DFPC can result in the Frank–Pryce configuration. Based on these phenomena, the authors successfully manipulated the configuration of the CLC droplets by alternately adding SDS and PVA. This work reveals the feasibility of fabricating LC biosensors using the CLC droplets.

Although the LC droplet with a spherical interface can endow the LC biosensor with high sensitivity by increasing the specific surface area, their stability is still unsatisfactory due to the fact that the optical texture will change with the volatilization of the surrounding aqueous. To increase the stability of the LC droplets, polymer shells are introduced to fully cover the LC phases in the droplets, resulting in the formation of the LC microcapsules [101,102,103]. The LC microcapsules show high sensitivity and satisfactory stability simultaneously, greatly expanding the application prospect of the LC droplets. In recent years, various LC microcapsules aimed at detecting different signals have been reported [104,105]. However, the fabrication of the LC microcapsules relies on interfacial polymerization, which remains complex.

## 3. Different Sensing Mechanisms

The LC biosensors are composed of LCs, alignment layers, and receptors, among which the receptors play the role of transmitters to pass the received biomolecule signals to the LCs, and the LCs act as the processors that can transform the biomolecule signals into the optical signals. For the LC processors, different processing mechanisms including birefringence, optical rotation, and reflection have been used, which have been introduced in the previous section. For the receptors, the signal transmission processes are based on various mechanisms such as immunoreaction, enzymatic reaction, and noncovalent interactions. In this section, we will introduce the signal transmission mechanisms in detail to explain the detection process of the LC biosensors theoretically.

### 3.1. Enzymatic Reactions

Enzymatic reactions have been used to realize the specific detection of the biomolecules [106]. LC biosensors based on the enzymatic reactions are usually fabricated by either immobilizing biological enzymes on the interfaces or dispersing them in the surrounding solution. During the enzymatic reaction, biological enzymes, which consistently out-perform the best man-made catalysts, are critical for accelerating the rates of numerous biological reactions. The key strategy used by enzymes to accelerate the reactions is stabilizing the chemical transition state of the zymolytes, and therefore reducing the reaction barrier [107]. The zymolytes can specifically bind the enzymes via the lock-and-key principle, in which the intermolecular interactions such as the electrostatic interaction, hydrophobic interaction, and topological interactions dominate the binding process, endowing the enzymatic reactions with satisfactory specificity [108]. Since diverse physiological activities are driven by the enzymatic reactions, the LC biosensors based on the enzymatic reactions have been supposed to be effective tools to diagnose diseases [109].

The reactions driven by different enzymes can produce different products, even if they have the same reaction zymolytes. For example, the degradation of glucose catalyzed by the glucose oxidase can generate H^+^ to reduce the pH value of the surrounding solution [110], while that catalyzed by the horseradish peroxidase leads to the generation of OH^−^, which in turn increase the pH value [111]. Enzyme inhibitors such as pepstatin, organophosphorus, and heavy metal ions can block the enzymatic reactions by decreasing the enzymatic activity [112]. Enzyme activators including the ethylene diamine tetraacetic acid, glutathione, and inorganic ions can enhance the enzymatic activity [113]. By subtly using the enzyme inhibitors and enzyme activators, the detection of the enzymatic reaction can be more flexible.

### 3.2. Immunoreactions

Immunoreactions are the defensive reactions made by the human body, holding the ability to remove the invading antigen [114]. Two different immunoreactions including nonspecific immunity and specific immunity have been deeply investigated. The nonspecific immunity is the first line of physiological defense against the antigen. The specific immunity can be further divided into humoral immunity and cellular immunity. Humoral immunity, also known as the antibody–antigen reaction, is the most common immunoreaction used in fabricating LC biosensors [115,116]. The excellent specificity of the antibody–antigen reactions is derived from the complementary spatial structures of the antibodies and antigens, showing promising application prospects in disease diagnosis and prevention. The antibody–antigen reactions can only occur when the molar ratios of the antibodies and antigens are in a certain range. Otherwise, the reaction rate and the reaction extent will be severely limited. Moreover, the antibody–antigen reactions are reversible that the immune complexes can be broken down into the antibodies and antigens again without influencing their physicochemical property and biological activity. Such reversibility promises the fabrication of the nondestructive LC biosensors based on the antibody–antigen reactions.

### 3.3. Hydrogen Bonds

Furthermore, hydrogen bonds, which mostly exist in the DNA double strands, have also been employed to achieve specific biomolecular detection. The DNA double strands derived from the hydrogen bonds possess two antiparallel nucleic acids assembled in the form of the right-handed double helixes [117]. The different basic groups in the oligonucleotides have been proven to be the key factors for forming hydrogen bonds. A tiny variation in the base sequence can lead to obvious differences in the hydrogen bond strength. The LC biosensors based on the complementary base pairing usually show outstanding specificity that can recognize different nucleic acids with only a 1-base mismatch [118]. Coordination interactions, which usually occur between the metal atoms and the organic ligands, are reliable candidates for imaging biomolecules. By depositing Ni nanoparticles on the substrate, the coordination interaction between the Ni nanoparticles and LC molecules can result in the ordered alignment of the LCs, which can be disrupted by the subsequently added biomolecules [119]. By modifying Au nanoparticles with thrombin via the coordination interaction, the detection of thrombin with high sensitivity is achieved [120]. The immobilization of the antigen at the LC–aqueous interface has been feasible using the coordination interaction between the antigen and the Ni-modified surfactant [90].

### 3.4. Nonspecific Interactions

Electrostatic interaction and hydrophobic interaction are the nonspecific interactions that have been widely employed in detecting biomolecules. The strength of the electrostatic interaction can vary with the electric density and molecular distance. Proteins and nucleic acids are the commonly charged biomolecules that can be detected by electrostatic interaction. Lai et al. have realized the detection of DNA using the electrostatic interaction between the electropositive surfactant and electronegative DNA [121]. Hu et al. successfully imaged antimicrobial peptide-induced membrane disruption based on the electrostatic interaction between the antimicrobial peptide and phospholipid [122]. The glucose oxidase (GOx) is immobilized on the LC biosensor by the electrostatic interaction to detect glucose [123]. It is worth mentioning that the electric densities of the biomolecules are related to the pH value of the surrounding solution, which can greatly influence the detection. For the amphoteric electrolytes such as proteins and polypeptides, isoelectric point (pI) is proposed to depict the relationship between the pH value and the electric density. As shown in the following equation,

(9) $$\mathrm{pI}=\frac{1}{2}\left({\mathrm{pK}}_{1}+{\mathrm{pK}}_{2}\right),\;$$

in which pK_1_ and pK_2_ are the dissociation constant of the acidic and alkaline group, respectively. When the pH value is smaller than pI, the biomolecules are electropositive. When the pH value is larger than pI, the biomolecules are electronegative. The biomolecules become electroneutral when the pH value is equal to pI. Consequently, the pH value should be strictly controlled in the LC biosensors based on the electrostatic interactions.

Hydrophobic interaction is the entropy-driven phenomenon that which the hydrophobic groups of the biomolecules aggregate together to keep away from the water phase, which is usually observed in physiological processes such as protein folding, membrane fusion, and food digestion [124]. The wetting behaviors of the groups are dependent on their surface free energy, i.e., when the surface free energy is larger than 72.8 J cm^−2^ (the surface free energy of water), the groups are hydrophilic. Otherwise, they are hydrophobic. Surfactants are amphiphilic molecules possessing charged hydrophilic groups and uncharged hydrophobic groups simultaneously, showing the ability to form the electrostatic interaction and hydrophobic interaction simultaneously, and therefore have been widely utilized in the LC biosensors. In general, current studies about the LC biosensors involve a variety of mechanisms that exhibit distinctly different formation causes and specificities. An in-depth understanding of these mechanisms can contribute to the efficient design of the LC biosensors.

## 4. Different Sensing Targets

### 4.1. Enzyme

Since enzymes can drive most of the cellular physiological activities, they have been regarded as important biomarkers for diagnosing diseases. Therefore, the detection of enzymes with both satisfactory specificity and sensitivity has become increasingly important in recent years. Based on the enzymatic reactions, LC biosensors aimed at detecting various enzymes including urease, cholylglycine hydrolase, protease, and carboxylesterases have been proposed, showing diversiform device structures and compositions. The specific recognition between the DNA aptamer and thrombin has also been utilized to design LC biosensors. Shown in Table 1 is a summary of the studies on the LC biosensors for detecting enzymes. 

In 2014, Liu et al. demonstrated a simple and label-free method for imaging urease activity using LC droplet patterns on solid surfaces [89]. The patterns are spontaneously obtained by dispersing stearic acid doped-LCs on the glass substrates. With the existence of the aqueous mixture of urease and urea, the enzymatic reaction between urea and urease produces ammonia and increases the pH value of the solution, leading to the deprotonation of the stearic acid. The deprotonated stearic acid can self-assemble at the LC–aqueous interface and induce the orientational transition of LC droplets from bipolar to radial. The LOD realized in this work is 1 μg mL^−1^. This work preliminarily realizes the fabrication of LC enzyme sensors, laying the foundation for later research.

Other enzymatic reactions participated by various enzymes have also been utilized to design LC biosensors. For example, Jannat et al. successfully fabricated an LC-based protease assay for naked-eye detection of protease activity [125]. Casein molecules are firstly cleaved by proteases into small peptide fragments in the assay. Then, trichloroacetic acid (TCA) is employed to separate and purify the generated peptide fragments. Finally, the peptide fragments are adsorbed on the substrate to disrupt the LC alignment, leading to the dark-to-bright transition of the optical image. By using the assay, the detection of 10 ng mL^−1^ protease has been feasible. Such an LC sensor can also be used for the detection of protease inhibition, which can be used for treating protease-related diseases [126]. When the protease inhibition is introduced into the sensor, the degradation reaction of casein catalyzed by the protease is blocked, and the optical image of the sensor remains dark.

**Figure 2:**
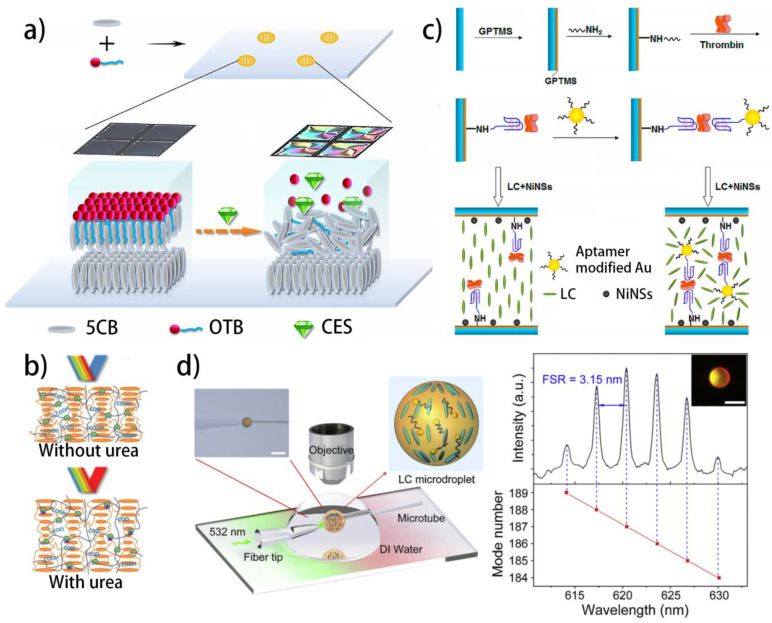
Different liquid crystal biosensors used for detecting enzymes: (**a**) detecting carboxylesterase using surfactant-doped liquid crystal biosensor [127]; reproduced with permission from Elsevier; (**b**) the sensing mechanism of the CLC/PAA IPN with urease detection property [129]; reproduced with permission from Wiley; (**c**) the fabrication and detection processes of the liquid crystal thrombin sensor based on Au nanoparticles [120]; reproduced with permission from the American Chemical Society; (**d**) schematic diagram of the experimental platform that measuring the LC penicillinase sensor by the WGM lasing [132]; reproduced with permission from Elsevier.

In 2019, an LC biosensor possessing carboxylesterases (CES) detection capacity was proposed simply based on the self-assembly of a cleavable surfactant, N-octadecyloxycarbonylmethyl-N, N, N-trimethylammonium bromide (OTB), on the LC–aqueous interface (Figure 2a) [127]. The OTB can trigger the vertical arrangement of the LC molecules by the hydrophobic interaction. Further, the CES can induce the hydrolysis of the OTB, resulting in the disruption of the LC arrangement as well as a dark-to-bright transition in the optical response. Based on the reaction between trypsin and poly-L-lysine (PLL), a simple and sensitive micro-capillary sensor for monitoring trypsin activity has also been achieved [128]. Trypsin levels as low as ~0.1 μg mL^−1^ can be detected.

Based on the Bragg reflection property of the CLCs, the biosensor arrays consisting of reactive CLCs and enzyme-immobilized polyacrylic acid (PAA) have been proposed to detect the urease activity in recent years. The detection result can be easily obtained by a color change. As shown in Figure 2b, Noh et al. functionalized the CLC/PAA interpenetrating polymer network (IPN) by urease via a simple EDC/NHS reaction [129]. When the IPN is in contact with urea, the enzymatic reaction between the urea and urease results in the increase in pH value and the deprotonation of PAA, which can expand the PAA chains and accordingly increase the helical pitches of the CLCs. Therefore, a redshift of the color can be observed by naked eyes. Such a CLC/PAA IPN can be further used to detect other physiological signals [130]. For example, by functionalizing the CLC/PAA IPN with phenylboronic acid, the detection of glucose can be realized. By treating the CLC/PAA IPN simply with KOH, the detection of divalent ions in the body fluid becomes feasible. As a result, this IPN platform opens the door for many interesting applications with numerous combinations of biomolecules and receptors, especially a great diversity of enzymatic reactions.

**Table 1:** Summarization of liquid crystal biosensors used for detecting enzymes.

Mesophase	Geometry	Receptor	Target	Sensing Mechanism	Refs.
**NLC**	LC droplets	Carboxylic acids	Urease/urea	The reaction between urea and urease produce ammonia to deprotonate carboxylic acids	[89]
**NLC**	LC–solid interface	Thrombin aptamer-functionalized AuNP	Thrombin	The specific combination between the thrombin and aptamer	[120]
**NLC**	LC–aqueous interface	Poly-L-lysine	Trypsin	The degradation of peptides under the catalysis of trypsin	[128]
**NLC**	LC–solid interface	Cholylglycine	Cholylglycine hydrolase	The degradation of cholylglycine under the catalysis of cholylglycine hydrolase	[131]
**NLC**	LC–solid interface	Casein	Protease	The degradation of casein under the catalysis of protease	[125]
**NLC**	LC–aqueous interface	OTB	Carboxylesterase	The degradation of OTB under the catalysis of carboxylesterase	[127]
**NLC**	LC–solid interface	Casein	Protease inhibition	Inhibiting the activity of protease by pefabloc	[126]
**NLC**	LC droplets	PBA	Penicillinase	The reaction between penicillinase and penicillin produce H^+^ to protonize PBA	[132]
**CLC**	CLC polymer	PAA	Urease	The reaction between urease and urea produce H^+^ to protonize PAA	[129]
**CLC**	CLC polymer	PAA	Urease/Glucose/ions	The reactions produce H^+^ to protonize PAA	[130]

Apart from the enzymatic reactions, the specific recognitions between enzymes and DNA aptamers, which also possess satisfactory specificity, have also been employed to detect enzymes. Aiming at detecting thrombin, a nickel nanosphere-doped LC sensor holding a sandwich system of aptamer/thrombin/aptamer-functionalized gold nanoparticles (Apt-AuNPs) has been fabricated by Zhao et al. [120]. As shown in Figure 2c, the aptamer, which can specifically bind thrombin, is immobilized on the substrate through the EDC/NHS reaction. The thrombin, which has two binding sites for the aptamer, can act as a bridge to link the monodispersed Apt-AuNPs together to make the aggregation. With the existence of thrombin, the LC molecules around the Apt-AuNPs show a chaotic arrangement under the influence of the steric hindrance provided by the aggregated Apt-AuNPs. Otherwise, the LC molecules arrange vertically under the influence of the coordination interaction brought by the doped nickel nanospheres, which have been proven by the authors in earlier work. Such a sensor can realize a LOD of 0.1 nM and a detection range of 0.1~100 nM. In 2017, Wei et al. also fabricated a nickel nanosphere-doped LC sensor, which can be used to visualize the enzymatic activity between cholylglycine hydrolase (CGH) and cholylglycine (CG) [131]. When CG (LOD: 10 pM) is introduced to the CGH immobilized gold surface, it will be decomposed into cholic acid and glycine, leading to the enhancement of the surface roughness, and therefore disrupting the Ni-induced arrangement of the LC molecules.

Moreover, to reduce the LOD of the LC enzyme sensor, the whispering gallery mode (WGM) lasing, which can effectively amplify the optical signal, has been employed to fabricate a novel LC biosensor with high sensitivity (Figure 2d) [132]. The sensor is fabricated by stabilizing fluorescent dye-doped LC droplets using 4′-pentyl-biphenyl-4-carboxylic acid (PBA). Due to the refractive index difference between the LCs and aqueous, the fluorescence emitted from the fluorescent dye can be trapped inside the droplets in the form of WGMs, showing a typical WGM lasing spectrum with high intensity. Such a WGM spectrum is closely related to the pH value, which can tune the refractive index of the droplets by changing the LC alignment. Furthermore, the reaction between penicillinase and penicillin can generate H^+^, and therefore change the pH value of the solution. As a result, the detection of penicillinase using the fluorescent dye-doped LC droplets sensor has been achieved. Due to the dual amplification effect of the LC molecules and WGMs, the LOD of 50 μM is realized. This work provides a new strategy for designing LC biosensors with high sensitivity.

### 4.2. Nucleic Acid

Nucleic acids are natural biopolymers of nucleotides. They can store, encode, transmit, and express genetic information, playing critical roles in a variety of physiological activities. The detection of DNA targets exhibits great significance in the field of clinical diagnostics, public health, food safety, and environmental monitoring, especially under the recent social condition that COVID-19 is threatening the health of people all over the world. Traditional nucleic acid detection techniques call for a complex fluorescent labeling process, which faces the problems of inherent photobleaching and fluorescence quenching. LC nucleic acid sensors can provide a label-free approach for the detection of nucleic acids with outstanding sensitivity, and have been studied extensively (Table 2). A variety of detection mechanisms including the hydrogen bond interaction, steric interaction, hydrophobic interaction, electrostatic interaction, and the Fenton reaction have been employed. Two different kinds of devices, based on the LC–solid interface and LC–aqueous interface, respectively, have been deeply investigated. The former one is promising in fabricating DNA microarrays, while the latter one holds higher sensitivity.

The first work of the LC nucleic acid sensor was reported by Kim et al. in 2005 [133]. They used a 19-mer oligoDNA of p53 tumor suppressor as a bioreceptor and its complementary partner as a target material and demonstrated the anchoring transition of the NLCs from a homeotropic to a random driven by the DNA hybridization. Specifically speaking, before the DNA hybridization, the LC molecules align vertically under the influence of the steric interaction between the LC molecules and the single-stranded DNA (ssDNA). In contrast, after the hybridization, the orientation of the LC molecules is disrupted by the double-stranded DNA (dsDNA) due to the increased steric hindrance.

Based on the pioneering work above, Lai et al. found that by adjusting the concentration and chain length of the oligonucleotide, the LC orientation can be directly disrupted by the ssDNA [134]. The concentration of ssDNA can influence the randomness of the LC molecules, and therefore tune the phase retardation of the incident light, resulting in the color variation of the transmitted light. When the DNA concentration increases from 0.5 μM to 5 μM, a color change from white to blue can be observed. As a result, the semiquantitative detection of the oligonucleotide, which has a good signal-to-noise ratio and is nondestructive to the oligonucleotides, is successfully realized.

Considering the explicit mechanism of ssDNA probes, dictating the orientation of LCs is still unclear. Shen et al. treated the glass substrate with different ssDNA solutions and compared the LC orientations on these surfaces [135]. It is found that the ssDNA probe with appropriate length and concentration can lead to the homeotropic orientation of LCs, otherwise the LC molecules align randomly due to either the inadequate orientation force or the excessive steric hindrance. The optimal condition for realizing homeotropic orientation is using an ssDNA probe containing 22 bases at a concentration of 20 nM. Once the complementary DNA is hybridized on the surface, the homeotropic orientation of the LCs becomes disrupted. Based on this optimal condition, the LOD of 0.1 nM has been realized, far smaller than the previous research.

**Figure 3:**
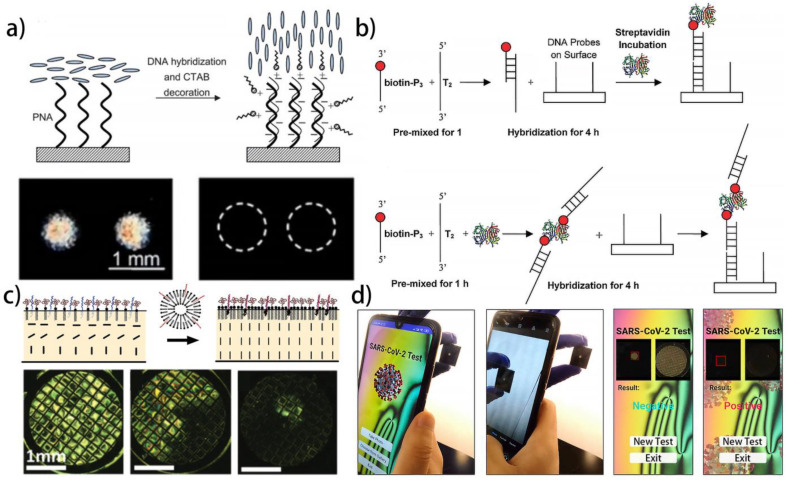
Different liquid crystal biosensors used for detecting nucleic acids: (**a**) schematic illustration of the liquid crystal nucleic acid sensor before and after detecting DNA [121]; reproduced with permission from Royal Society of Chemistry; (**b**) two strategies of introducing streptavidin to the liquid crystal nucleic sensor to increasing the contrast [136]; reproduced with permission from the American Chemical Society; (**c**) DNA hybridization-mediated liposome fusion at the aqueous–liquid crystal interface [137]; reproduced with permission from Wiley; (**d**) liquid crystal-based naked-eye home detection kit for detecting RNA of the SARS-CoV-2 [141]; reproduced with permission from Elsevier.

Although the interaction between oligonucleotides and LC molecules have been deeply investigated to fabricate LC nucleic acid sensor, the reproducibility of the optical image is still unsatisfactory. This is due to the steric interaction provided by oligonucleotide weakness. In order to address this problem, Lai et al. introduced surfactants into the DNA solution (Figure 3a) [121]. The surfactants can form complexes with DNA by electrostatic interaction and assist in the LC alignments. The electroneutral peptide nucleic acids (PNA) probes are immobilized on the substrate to capture DNA targets. In the presence of the DNA targets, the substrate is electronegative and can further absorb the electropositive surfactant cetyl trimethylammonium bromide (CTAB). As a result, a PNA/DNA/CTAB complex is formed and the LC molecules align perpendicularly under the influence of CTAB. The LOD realized by this strategy is 1 μM. Further, they also introduced streptavidin into the DNA solution, aiming at increasing the contrast of the optical images [136]. As shown in Figure 3b, two different approaches to introducing streptavidin to the system are studied and compared. It is found that by premixing the biotin-labeled DNA targets with streptavidin, branched DNA is formed and the steric interaction provided by DNA is greatly enhanced, resulting in a clear LC signal with high contrast.

**Table 2:** Summarization of liquid crystal biosensors used for detecting nucleic acids.

Mesophase	Geometry	Receptor	Target	Sensing Mechanism	Refs.
**NLC**	LC–solid interface	DNA aptamer	DNA	The hydrogen bond interaction between DNA single strands	[133]
**NLC**	LC–solid interface	DNA aptamer	DNA	The quantification of DNA concentrations through the interference colors of LCs	[134]
**NLC**	LC–solid interface	CTAB/PNA	DNA	The electrostatic interaction between DNA and CTAB, and the hydrogen bond interaction between DNA single strands	[121]
**NLC**	LC–solid interface	DNA streptavidin complex	DNA	The hydrogen bond interaction between DNA and DNA streptavidin complex	[136]
**NLC**	LC–solid interface	DNA aptamer	DNA	The LC alignment is related to the concentration and chain length of the DNA	[135]
**NLC**	LC–aqueous interface	OTAB/DNA	DNA	The electrostatic interaction between DNA and OTAB, and the hydrogen bond interaction between DNA single strands	[118]
**NLC**	LC–aqueous interface	OTAB/DNA	DNA	The electrostatic interaction, hydrogen bond interaction, and hydrophobic interaction	[88]
**NLC**	LC–aqueous interface	PEG-lipid monolayer decorated with DNA	Bulk phase liposomes decorated with DNA	DNA hybridization-mediated liposome fusion	[137]
**NLC**	LC–aqueous interface	DNA-lipids	DNA	The hydrogen bond interaction between DNA single strands	[138]
**NLC**	LC–aqueous interface	DTAB/DNA	DNAs of bacterium Erwinia carotovora and fungi Rhazictonia solani	The electrostatic interaction between DNA and DTAB, and the hydrogen bond interaction between DNA single strands	[139]
**NLC**	LC–aqueous interface	OTAB	The Fenton reaction of DNA	The electrostatic interaction and Fenton reaction	[140]
**NLC**	LC–aqueous interface	DTAB	RNA of SARS-CoV-2	The electrostatic interaction between DNA and DTAB, and the hydrogen bond interaction between DNA and RNA	[141]

In the LC nucleic acid sensors mentioned above, the ssDNA probes are modified on the solid substrate. Such sensors based on the LC–solid interface are promising in fabricating DNA microarrays, which allow the simultaneous detection of a large amount of DNA targets at one time. However, the LODs of the sensors are severely limited due to the inadequate contact between the ssDNA probes and ssDNA targets. To solve this problem and enhance the sensitivity, another kind of LC nucleic acid sensor, which is based on the LC–aqueous interface, has also been proposed.

In the work reported by Price et al., the adsorption of octadecy trimethyl ammonium bromide (OTAB) surfactant to the LC–aqueous interface leads to the homeotropic alignment of the LC molecules [118]. While the subsequent combination of ssDNA and surfactant leads to the desorption of OTAB and results in a homeotropic-to-parallel orientation transition of the LCs. Further treating the interface by the solution containing the ssDNA complement can cause a second change in the LC alignment. This is because the hydrogen bond interaction between the complementary ssDNA is stronger than the electrostatic interaction between the ssDNA and OTAB. The OTAB can reassemble at the LC–aqueous interface [88]. Such a strategy can realize a LOD of 50fM, far lower than the aforementioned work by four orders of magnitude.

In addition, DNA hybridization at the LC–aqueous interface has been employed to mediate the liposome fusion, which shows great significance to the study of the membrane fusion mechanisms (Figure 3c) [137]. Specifically speaking, the micelles composed of ssDNA and polyethylene glycol (PEG)-modified liposome are firstly obtained, in which the PEG acts as the steric barrier to hinder the liposome from spontaneous fusion. When the micelles are introduced to the LC–aqueous interface, the liposome and ssDNA in the micelles can self-assemble at the interface under the force of hydrophobic interaction. In this state, the LC molecules align in parallel, indicating that the density of the liposome at the interface is too low to provide enough orientation force. Then, the DNA hybridization process between complementary ssDNA within bulk phase liposomes and the liposome-assembled interface is exploited to induce hybridization-mediated liposome fusion, which induces the strain within the liposome bilayer and promotes the lipid mixing at the interface. In this state, the LC molecules align vertically, indicating the density of the liposome at the interface is enough to trigger the homeotropic orientation. This work shows promise for high throughput screening of membrane fusion. Such a phenomenon about the DNA–lipid complex has been further verified by Zhou et al. in subsequent work [138].

With the development of the LC nucleic acid sensors, their applications in disease diagnosis have also aroused great attention. Khan et al. reported an LC-based DNA biosensor for pathogen detection. They successfully realize the label-free detection of the genomic DNAs of the bacterium *Erwinia carotovora* and the *fungi Rhazictonia solani* with high specificity and sensitivity [139]. Kim et al. achieved the detection of DNA damage by combining the LC nucleic acid sensor with the Fenton reaction [140]. The adsorption of ssDNA onto the surfactant-laden LC–aqueous interface can lead to a planar orientation of the LCs. However, the Fenton reaction introduced in the sensor can produce reactive oxygen species, which possess strong oxidizing properties and can break the ssDNA to change the LC orientation. Recently, an LC sensor is used for the ultrasensitive and selective detection of SARS-CoV-2 with a LOD of 30 fM [141]. Based on this sensor, an LC diagnostic kit and a smartphone-based application are further developed via the image-based machine learning strategy to enable automatic detection (Figure 3d). This work provides an alternative approach for reliable self-test of SARS-CoV-2 at home, holding important social value under the recent pandemic condition.

### 4.3. Protein and Amino Acid

Proteins, as the key working units for cells and tissues in the human body, dominate a variety of physiological activities ranging from storage and metabolism of energy to immunoreaction. They are composed of diverse amino acids, possessing complex spatial structures such as α-helix and β-sheet. Believing that the abnormal expression of proteins is usually closely related to diseases, the precise detection of proteins is of great significance. Based on the different receptors including antibody–antigen, Au nanoparticle (AuNP), polyelectrolyte, surfactant, and nucleic acid, various LC protein sensors have been proposed successively. Efforts have also been devoted to investigating the quantitative measurement of proteins, as well as the molecular dynamics mechanism behind protein detection. Shown in Table 3 is a summary of the studies on the LC biosensors for detecting proteins.

Hartono et al. first realized the real-time detection of specific protein–protein binding using protein-decorated LC interface [90]. The self-assembly of the amphipathic coordination compound 1,2- dioleoyl-sn-glycero-3-{[N(5-amino-1-carboxypentyl) iminodiacetic acid] succinyl} (nickel salt) (DOGS-NTA-Ni) at the LC–aqueous interface creates a surface capable of immobilizing histidine-tagged ubiquitin via the coordination between Ni^2+^ and histidine. When the surfaces containing ubiquitin are exposed to anti-ubiquitin antibodies, the protein–protein binding events can trigger the orientational transition of LCs within seconds. The LOD realized in this work is 5.2 μg mL^−1^. However, the immobilization of histidine-tagged ubiquitin takes 2 h, which severely limits the detection efficiency of the sensor.

**Figure 4:**
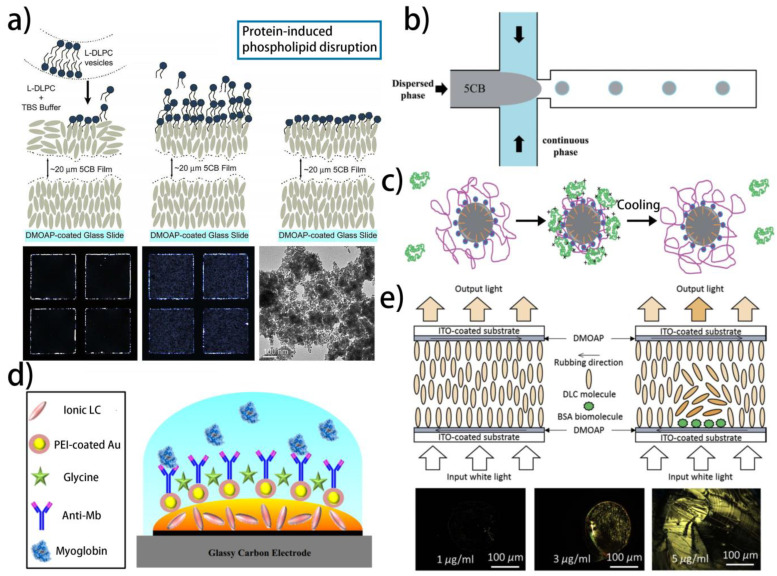
Different liquid crystal biosensors used for detecting proteins: (**a**) imaging the protein-induced phospholipid disruption using a liquid crystal protein sensor [142]; reproduced with permission from Elsevier; (**b**,**c**) the fabrication process and reversible detection property of the liquid crystal protein sensor based on the thermal responsive PNIPAAm [147]; reproduced with permission from Royal Society of Chemistry; (**d**) schematic representation of liquid crystal immunosensor sensor based on the ionic liquid crystals [150]; reproduced with permission from Elsevier; (**e**) schematic illustration of the dye liquid crystal-based biosensing platform [154]; reproduced with permission from Elsevier.

In the same year, they also realized the imaging of the phospholipid monolayer disruption triggered by the protein-coated nanoparticles using an LC sensor (Figure 4a) [142]. In body fluids, the adsorption of protein on the surface of nanoparticles is common, and cells are likely to interact with protein-coated nanoparticles instead of bare nanoparticles. In order to simulate this process, protein-coated gold nanoparticles (AuNPs) are fabricated by incubating AuNPs and proteins together. Then, the introduction of the protein-coated AuNPs to the phospholipid monolayer-coated LC–aqueous interface can lead to the disruption of phospholipid and the reorientation of the LCs, which is caused by the hydrophobic interaction between the protein and phospholipid. This work provides a new approach to studying the potential cytotoxicity of nanomaterials. By replacing the AuNPs with antimicrobial peptide, the simulation of antimicrobial peptide-induced membrane disruption is also achieved based on the electrostatic interaction between the antimicrobial peptide and phospholipid [122]. Based on the redox reaction between HAuCl_4_ and L-DOPA, which is generated by the hydroxylation of tyrosine catalyzed by tyrosinase, the AuNPs can be obtained to change the orientation of the LCs, and therefore realize the detection of tyrosine [143].

Park group has reported a series of works that fabricated LC protein sensors using polyelectrolytes as the receptors. For example, they fabricated the LC droplets coated with the polyelectrolyte PAA-b-LCP (LCP: poly(4-cyanobiphenyl-4-oxyundecylacrylate)) [144]. The PAA-b-LCP acts as the alignment agent with its LCP chain contacts with the LC while its PAA tail contacts with water. When detecting proteins such as lysozyme and bovine serum albumin, the LC orientation in the sensor can be changed under the influence of electrostatic interaction. This orientation transition is strongly dependent on the electrostatic states of the proteins and PAA chains and is related to the pH value of the solution. By decorating the LC–aqueous interface with PAA-b-LCP, the detection of several proteins can also be realized through the homeotropic to planar orientation transition of the 5CB [145]. The sensor has been used to detect proteins of human urine from a patient having albuminuria with a detection limit as low as 0.032 mg mL^−1^.

Moreover, by utilizing another polyelectrolyte PNIPAAm-b-LCP (PNIPAAm: poly(N-isopropyl acrylamide)), which possesses temperature-responsive property, the homeotropic-to-planar transition induced by the protein adsorption becomes reversible. The protein detections using LC–aqueous interfaces decorated with PNIPAAm-b-LCP, as well as using LC droplets coated with PNIPAM-b-LCP and SDS, have been successfully demonstrated (Figure 4b,c) [146,147]. Yang et al. further studied the LC protein sensors based on the polyelectrolytes and realized the detection of proteins using quarternized poly-4-vinylpyridine (QP4VP, cationic polyelectrolyte) and polystyrenesulfonate (PSS, anionic polyelectrolyte), respectively [148]. Although the advantages of the polyelectrolyte-based LC protein sensors, such as high sensitivity, simple preparation process, and reversibility have been verified by a large number of efforts, the poor selectivity of such sensors remains to be improved.

With the development of ionic liquids, ionic liquid crystals (ILCs), born at the interface of ionic liquids and liquid crystals, have aroused great attention. ILCs are composed of anions and cations, holding the orientation of LCs and the electrical conductivity of ions at the same time. They have been widely used in the field of ionic conductive materials, organic reaction agents, and functional nanomaterials. Zapp et al. fabricated an LC protein sensor based on the ILC Br-Py and the silane-modified AuNP (AuNP-Si4Pic^+^Cl^−^) [149]. The Br-Py in the sensor can amplify the protein signal and transform it into an electric signal. Further, the AuNP-Si4Pic^+^Cl^−^ plays the role of a protein receptor. To fabricate the sensor, the Br-Py solution was dropped onto the glassy carbon electrode (GCE), followed by dripping a film of AuNP-Si4Pic^+^Cl^−^ onto the Br-Py coated GCE. Next, the GCE was incubated with anti-troponin T monoclonal antibodies (ab-cTnT) and troponin T (cTnT) in succession, and the electrochemical behavior was studied by cyclic voltammetry and electrochemical impedance spectroscopy. The immune complexes composed of ab-cTnT and cTnT can block the redox probe electron transfer process on the electrode surface, and therefore change the electrochemical behavior. Such a strategy can realize the LOD of 0.076 ng mL^−1^ and a linear range between 0.1–0.9 ng mL^−1^.

In order to further increase the stability of the ILC sensor, the authors then replaced the AuNP-Si4Pic^+^Cl^−^ with a film of polyethyleneimine-coated gold nanoparticles (AuNP-PEI), which could bind the antibody by the covalent bond (Figure 4d). The proposed method for antigen detection is based on the voltammetric suppression of the Br-Py signal when the immunosensor is incubated with the antigen. The immunosensor shows a good linear relationship between the electrochemical inhibition response and the concentration of the antigen over the range of 9.96–72.8 ng mL^−1^ with a detection limit of 6.29 ng mL^−1^, indicating the good sensitivity of the sensor [150]. These efforts in the development of the ILC protein sensor greatly increase the diversity of the LC biosensors.

In recent years, the quantitative detection of proteins has been investigated in-depth to lay the groundwork for clinical applications. Various strategies have been employed to realize quantitative detection. For instance, Das et al. reported the detection of hemoglobin, bovine serum albumin, and lysozyme proteins via the lipopolysaccharide (LPS) coated LC–aqueous interface [151]. The concentrations of the proteins are determined by the optical retardation of the LCs, which can influence the interference color of the optical images. Zhang et al. observed the homeotropic-to-tilted alignment transition of LCs induced by the specific binding event between cecropin B and anti-cecropin B antibodies [152]. The average gray-scale intensities of the optical images are calculated to quantify the cecropin B concentrations. Furthermore, Concellon et al. fabricated a Janus CLC droplet consisting of immiscible CLCs and fluorocarbon oils (FCs), using boronic acid polymeric as the surfactant [153]. Owing to the asymmetric density of the CLCs and FCs, the Janus droplets can self-align on a horizontal surface under the influence of gravity, ensuring a normal reflection of the light. The boronic acid polymeric, which is decorated with IgG antibodies, can specifically recognize the antigens on the bacteria via the immunoreaction, and the concentration of the bacteria can be determined by the blueshift of the reflection band.

Moreover, dye liquid crystals (DLCs) and dual-frequency liquid crystals (DFLCs) have also been used for realizing quantitative detection. Wu et al. have proposed a DLC-based biosensing strategy for quantitative protein assay in 2018 [154]. As exhibited in Figure 4e, the DLC utilized for fabricating the sensor is an azobenzene LC, exhibiting unique optical anisotropy and dichroic absorption. The orientation transition of the DLC molecules driven by the bovine serum albumin immobilization can lead to the relative loss in the transmittance, and provide a reliable sensing platform for the quantitative analysis of biomolecules. In 2019, a DFLC-based biosensor with frequency-dependent dielectric properties was developed to quantify bovine serum albumin [155]. The DFLC used in the sensor exhibits positive dielectric anisotropy at low frequencies and negative dielectric anisotropy at high frequencies. The difference in dielectric permittivity between the high- and low-frequency regimes is correlated to the concentration of bovine serum albumin. However, the detailed mechanism behind the dielectric permittivity variation is still not fully understood. Further efforts remain to be devoted to studying the detection mechanism at the molecular scale.

Nucleic acids have also been proven to be the ideal receptors for proteins with high specificity. The DNA aptamer with alpha-synuclein binding property has been modified on the glass substrate by Yang et al., followed by the incubation of alpha-synuclein on the substrate and the fabrication of LC cell [156]. When the concentration of alpha-synuclein is higher than 50 nM, the random arrangement of LC can be observed by POM. Considering that alpha-synuclein is closely related to Parkinson’s Disease (PD), this sensor provides an alternative approach for the early PD diagnosis. Aiming at further increasing the sensitivity of the sensor, the DNA aptamer is directly absorbed on the CTAB-laden LC–aqueous interface [157]. In the presence of alpha-synuclein, the DNA aptamer specifically binds with alpha-synuclein and releases CTAB, leading to the homeotropic alignment of LCs. Such a sensor can realize the detection of alpha-synuclein with a LOD as low as 10 pM.

**Figure 5:**
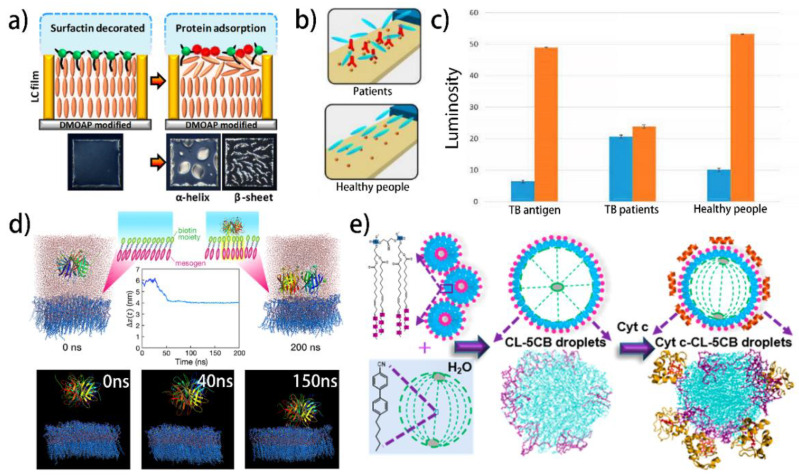
Advanced applications of liquid crystal protein sensors: (**a**) liquid crystal biosensor enabled identification of secondary structure of proteins [158]; reproduced with permission from the American Chemical Society; (**b**,**c**) the clinical diagnosis using the liquid crystal tuberculosis sensor [159]; reproduced with permission from the American Chemical Society; (**d**) molecular dynamics simulation study of protein binding at the liquid crystal–aqueous interfaces [160]; reproduced with permission from the American Chemical Society; (**e**) unveiling the lipid–protein interactions that drive the reorientation at the LC–droplet interface using the atomistic simulations [161]; reproduced with permission from the American Chemical Society.

Verma et al. reported the identification of the secondary structure of proteins using a surfactin-laden LC–aqueous interface (Figure 5a) [158]. In the absence of proteins, the surfactin can promote the homeotropic alignment of the LCs. While in the presence of proteins, several noncovalent interactions such as electrostatic, hydrogen bonding, and hydrophobic interaction can lead to the reorientation of the LCs, giving rise to the bright optical appearances under POM. The shapes of bright spatial patterns are directly associated with the native secondary conformations of proteins such as β-sheet and α-helix.

The clinical application of the LC protein sensors has been demonstrated by Kim et al. [159]. As shown in Figure 5b,c, tuberculosis antibody (ab-TB) is immobilized on the substrate via the simple EDC/NHS reaction. The clinical serum samples are incubated on the substrate. The specific binding of ab-TB and TB only occurred in the clinical specimens from TB patients, leading to the disruption of the LC orientation. The detection accuracy for the TB patients is 76%, and for healthy people is 91%. The accuracy of this strategy can be effectively increased by several repetitive tests for the diagnosis of TB. However, the detection accuracy for the latent TB patient is only 25%, which is far away from the effective clinical diagnosis. This is because there is not enough ab-TB in the latent TB patient serum to induce the LC reorientation. Further increase in the sensitivity of the sensor by optimizing the structure can be a feasible approach to solving this problem.

Aiming at providing guidance to the future molecular-level design of the LC protein sensors, efforts have been dedicated to the molecular dynamics (MD) simulation of the detection process. Watanabe et al. studied the streptavidin-biotin-LC system via the simulation (Figure 5d) [160]. Streptavidin is a protein secreted by the streptomycete, possessing four peptide chains that can bind biotin specifically. The specific recognition between streptavidin and biotin has been widely used in antibody detection, affinity chromatography, and fluorescence labeling. Two different molecular models including a biotin-conjugated mesogenic molecule and a biotin-free mesogenic molecule are employed to perform the all-atom MD simulation. The simulation result reveals that the binding of streptavidin to the biotin mesogenic molecule is significantly stronger than that to the biotin-free mesogenic molecule. The streptavidin binding can slightly increase the tilt of the biotin-conjugated mesogenic molecules by ~10°, as well as impact the diffusion properties of the water molecules near the biotin mesogenic molecules. This work can advance the understanding of the molecular-level phenomena involved in the protein detection process using the LC protein sensor.

Recently, the lipid–protein interactions at the interface of the LC droplets are investigated at the nanoscale via the atomistic simulation (Figure 5e) [161]. It is noted that in the cardiolipin (CL)-LC system, the acyl chains of CL not only lie at the periphery of the droplet but also penetrate the droplet, leading to the radial orientation of the LC. In the cytochrome c (Cyt c)-CL-LC system, the acyl chains show a tendency of migrating to the periphery, implying that the CL tails undergo rearrangement in the presence of Cyt c. It is also found that the binding of Cyt c to the interfacial CL does not trigger the unfolding of the protein, indicating the potential application of this system in the field of nondestructive tests. The underlying interactions revealed in this study will contribute to the rational design of the LC protein sensors.

**Table 3:** Summarization of liquid crystal biosensors used for detecting proteins.

Mesophase	Geometry	Receptor	Target	Sensing Mechanism	Refs.
NLC	LC–aqueous interface	DOGS-NTA-Ni and histidine-tagged ubiquitin	Anti-ubiquitin antibody	Immunoreaction	[90]
NLC	LC–aqueous interface	Phospholipids	Protein-coated AuNPs	The hydrophobic interaction between phospholipids and protein	[142]
NLC	LC droplets	PAA-b-LCP	Lysozyme and BSA	The electrostatic interaction between proteins and PAA	[144]
NLC	LC–aqueous interface	PAA-b-LCP	Lysozyme, BSA, lactalbumin, and insulin	The electrostatic interaction between proteins and PAA	[145]
NLC	LC–aqueous interface	PNIPAAm-b-LCP	Lysozyme, BSA, hemoglobin, and chymotrypsinogen	The electrostatic interaction between proteins and PNIPAAm	[146]
NLC	LC droplets	PNIPAAm-b-LCP	BSA, lysozyme, hemoglobin, and chymotrypsinogen	The electrostatic interaction between proteins and PNIPAAm	[147]
NLC	LC–aqueous interface	Phospholipids	Antimicrobial peptides	The electrostatic interaction between phospholipids and antimicrobial peptides	[122]
ILC	LC–solid interface	AuNP-Si4Pic^+^Cl^−^ and ab-cTnT	cTnT	Immunoreaction	[149]
ILC	LC–solid interface	AuNP-PEI and ab-Mb	Myoglobin	Immunoreaction	[150]
NLC	LC droplets	QP4VP and PSS	Hemoglobin and BSA	The hydrophobic interaction between polyelectrolytes and protein	[148]
NLC	LC–solid interface	HAuCl_4_	Tyrosine	Enzymatic reaction	[143]
NLC	LC–aqueous interface	Lipopolysaccharide	Hemoglobin, BSA, and Lysozyme	The hydrophobic interaction between lipopolysaccharide and protein	[151]
NLC	LC–solid interface	Anti-TB	TB antigen	Immunoreaction	[159]
NLC	LC–solid interface	Anti-cecropin B	Cecropin B	Immunoreaction	[152]
DLC	LC–solid interface	No receptor	BSA	BSA directly changes the LC orientation	[154]
DFLC	LC–solid interface	No receptor	BSA	BSA directly changes the LC orientation	[155]
NLC	LC–aqueous interface	Biotin	Streptavidin	The combination of biotin and streptavidin	[160]
NLC	LC–aqueous interface	Surfactin	Proteins with different secondary conformations	The electrostatic interaction between surfactin and proteins	[158]
NLC	LC–solid interface	DNA aptamer	Parkinson’s Disease related alpha-synuclein	The specific binding of protein to DNA aptamer	[156]
NLC	LC–aqueous interface	CTAB/DNA aptamer	Parkinson’s Disease related alpha-synuclein	The specific binding of protein to DNA aptamer	[157]
NLC	LC droplets	Cardiolipin	Cytochrome c	The electrostatic interaction between cardiolipin and cytochrome c	[161]

### 4.4. Other Target

#### 4.4.1. Glucose

**Figure 6:**
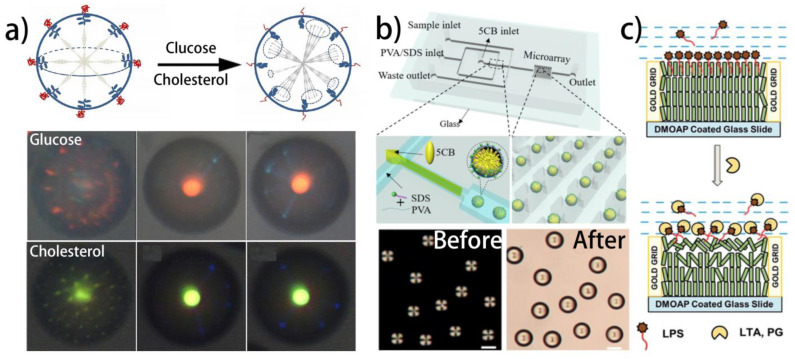
Different liquid crystal biosensors used for detecting glucose, cholesterol, Lipopolysaccharides, and bile acids: (**a**) pH responsive liquid crystal droplets used for detecting glucose and cholesterol [163]; reproduced with permission from the American Chemical Society; (**b**) liquid crystal lipopolysaccharides sensor based on the interaction of lipopolysaccharides with peptidoglycan and lipoteichoic acid [171]; reproduced with permission from Elsevier; (**c**) the LC biosensor for rapid and precise recognition of the interaction of LPS with PG and LTA [174]; reproduced with permission from Royal Society of Chemistry.

Apart from the essential biomolecules mentioned above, some other molecules, such as glucose, cholesterol, endotoxin, and bile acid, also play important roles in vital activities. Various LC biosensors have been proposed to realize their detection (Table 4). Kim et al. first realized the detection of glucose using the PAA-b-LCP-laden LC droplets [162]. Glucose oxidase (GOx) is covalently immobilized to the PAA chains at the droplet surface via the simple EDC/NHS reaction. With the existence of glucose, the enzymatic reaction between GOx and glucose can generate H^+^, leading to the protonation of the PAA and the swelling of the PAA-b-LCP. As a result, the reorientation of the LC molecules from radial to bipolar can be observed under POM. The LOD realized in this work is 0.03 mM. By covalently immobilizing cholesterol oxidase (ChO) to the PAA-b-LCP-laden CLC droplets, the detection of cholesterol using a CLC biosensor at a concentration as low as 2.5 μM has been achieved (Figure 6a) [163].

Moreover, the LC glucose sensor based on the LC–aqueous interface has also been proposed. Zhong et al. reported a highly sensitive and selective glucose sensor based on the ultraviolet-treated NLCs, which exhibit amphiphilic properties and can be used as the surfactant [164]. Submerging the sensor in a glucose solution can trigger an optical response from dark to bright. In later work, Khan et al. developed another sensor by coating LCs with a monolayer of mixed polymer brushes containing PAA-b-LCP and QP4VP-b-LCP [123], in which the QP4VP-b-LCP can immobilize GOx through electrostatic interactions without the aid of coupling agents, greatly simplifying the fabrication process. To obtain a more visualized optical signal, a photonic interpenetrating polymer network (IPN) structure consisting of intertwined CLCs and cationic polyelectrolyte networks has been demonstrated [165]. The enzymatic reaction of GOx with glucose can cause a redshift of the reflected light color.

Apart from utilizing GOx, some other strategies, such as bienzyme strategy and enzyme-free strategy, are also reliable in designing LC glucose/cholesterol sensors. In the work declared by Khan et al., GOx and horseradish peroxidase (HRP) are co-immobilized on the PAA-b-LCP-laden LC–aqueous interface [166]. With the existence of glucose, the enzymatic reaction between GOx and glucose generates H_2_O_2_ and gluconic acid. Then, H_2_O_2_ is degraded to OH^−^ under the catalysis of HRP, increasing the pH value of the solution and changing the orientation of the LCs. By replacing the GOx with ChO, such a bienzyme strategy can also be employed to realize the detection of cholesterol with high sensitivity [167]. The enzyme-free detection of glucose has been realized by coupling boronic acid at the poly (styrene-b-acrylic acid) (PS-b-PAA)-laden LC–aqueous interface [168], as well as the PS-b-PAA-laden LC droplets [169]. The specific reaction between glucose and boronic acid, which can generate H^+^, is successfully imaged by observing the LC orientation.

#### 4.4.2. Bile Acids

Bile acids are the amphipathic products of the cholesterol metabolism that aid in digestion and signal transduction in the digestive system, and have been used as the indicators of hepatobiliary and intestinal diseases [170]. They can act as the emulsifier to remove lipids from the fatty food and promote the formation of micelles, accelerating the uptake of fatty food by intestinal mucosal cells. In order to realize the detection of bile acids, Niu et al. developed an LC bile acid sensor based on the SDS surfactant-laden LC droplets [171]. A radial-to-bipolar transition of LC molecules can be triggered by the competitive reaction of bile acids and SDS, promising a low-cost, simple, and fast approach for detecting bile acids with a LOD of 5 μM (Figure 6b). Then, Kim et al. further studied the effects of the pH value and chain length of the surfactant on the detection of bile acids [172]. It has been proven that with the increase in the pH value, the electrostatic repulsion between the SDS and bile acids will increase, and the competitive adsorption of the bile acids will be impeded. As a result, the LOD of the sensor will be higher. As the chain length of the surfactant increases, the competitive adsorption of the bile acids will be more difficult at the more hydrophobic surfactant-laden interface, which also increases the LOD.

Recently, the quantitative measurement of bile acids has been achieved by a novel monodisperse LC droplet-based polydimethylsiloxane (PDMS) microchip [173]. The flow-focusing element is employed to generate monodisperse LC droplets stabilized by PVA/SDS. The droplet trapping structure is designed for the entrapment of the droplets. By monitoring the collapse of 5CB droplets caused by the bile acids, the percentage of the collapsed 5CB droplets is calculated to quantitatively analyze the detection results.

**Table 4:** Summarization of liquid crystal biosensors used for detecting other targets.

Mesophase	Geometry	Receptor	Target	Sensing Mechanism	Refs.
NLC	LC droplets	Glucose oxidase modified PAA	Glucose	Enzymatic reaction	[162]
CLC	LC droplets	Glucose oxidase and cholesterol oxidase modified PAA	Glucose and cholesterol	Enzymatic reaction	[163]
NLC	LC–aqueous interface	Glucose oxidase	Glucose	Enzymatic reaction	[164]
NLC	LC–aqueous interface	Glucose oxidase-immobilized QP4VP	Glucose	Enzymatic reaction	[123]
CLC	CLC polymer	Glucose oxidase	Glucose	Enzymatic reaction	[165]
NLC	LC–aqueous interface	Glucose oxidase and horseradish peroxidase	Glucose	Enzymatic reaction	[166]
NLC	LC–aqueous interface	Cholesterol oxidase and horseradish peroxidase	Cholesterol	Enzymatic reaction	[167]
NLC	LC-aqueous interface	3-aminophenyl boronic acid	Glucose	The specific reaction between 3-aminophenyl boronic acid and glucose	[168]
NLC	LC droplets	3-aminophenyl boronic acid	Glucose	The specific reaction between 3-aminophenyl boronic acid and glucose	[169]
NLC	LC–aqueous interface	Peptidoglycan and lipoteichoic acid	bacterial endotoxin	HydrophobicInteraction	[174]
NLC	LC–solid interface	DNA aptamer	Lipopolysaccharides	The specific reorganization of lipopolysaccharides using DNA	[175]
NLC	LC droplets	SDS	Bile acid	Hydrophobic interaction	[171]
NLC	LC–aqueous interface	Surfactants with different chain lengths	Bile acid	Hydrophobic interaction	[172]
NLC	LC droplets	SDS	Bile acid	Hydrophobic interaction	[173]

#### 4.4.3. Lipopolysaccharides

Lipopolysaccharides (LPS), also known as endotoxins, have been proven as a type of pyrogen that can be found in Gram-negative bacteria. They possess strong physiological toxicity that can lead to fever, inflammation, sepsis, and tissue damage, even in small quantities. Therefore, the detection of endotoxins with high sensitivity has been paramount. Considering that the interactions of various bacterial cell membrane components such as peptidoglycan (PG) and lipoteichoic acid (LTA) with LPS can result in diverse consequences on the toxicity, an LC biosensor has been designed for rapid and precise recognition of the interaction of LPS with PG and LTA (Figure 6c) [174]. LPS is utilized as the surfactant at the LC–aqueous interface to induce a homeotropic alignment of the LCs. The LC reorientation can be observed with the existence of PG and LTA. In addition, PG and LTA show different binding affinities towards LPS, and the LC reorientation is found to vary obviously from one to another, which can be conveniently quantified by measuring the optical retardance. An et al. have proposed an LC biosensor using LPS-specific ssDNA aptamers as LPS-selective probes [175]. In the presence of LPS, the combination of LPS and ssDNA can lead to the random orientation of LC molecules. By treating the sensor with an acid solution (pH < 5), the LC orientation can be fully recovered. Such a reusable strategy further expands the application prospects of the LC LPS sensors.

## 5. Conclusions and Outlook

The LCs show impressive stimuli-responsive properties and optical modulation properties, with applications over diverse areas. Integrating the LCs and receptors together, the LC biosensors aimed at detecting various biomolecules have been widely explored. Compared with the traditional biosensing technologies, the LC biosensors hold the advantage of being simple and efficient, as well as the visualized output signals that can be directly observed by the naked eye using optical techniques. Up to now, mesophases such as NLCs and CLCs have been employed to fabricate the LC biosensors. The former exhibits variable optical signal under POM via birefringence, and the latter possesses tunable reflection color through Bragg reflection. ILCs, as the novel materials born at the interface of the ionic liquids and LCs, have also been used in biosensing, achieving the LC biosensors with satisfactory electrical properties. The geometries of the sensors, which confine the LCs in the designated spaces to impose the specific LC alignments, can greatly influence the detection property of the biosensors. The LC–solid interface holds superiority in achieving sensing microarrays, while the LC–aqueous interface can permit the biosensors with higher sensitivity. For the LC droplets with spherical geometry, the sensitivity can be further improved despite the unsatisfactory stability. Moreover, various mechanisms such as immunoreaction, enzymatic reaction, and noncovalent interactions have been employed to bind biomolecules in biosensors. The studies on these mechanisms can contribute to the efficient design of the LC biosensors. Based on the diversified mesophases, geometries, and mechanisms, the detection of biomolecules such as enzymes, proteins, nucleic acids, and glucose using the LC biosensors has been investigated in-depth.

In spite of the rising research enthusiasm for LC biosensors, there remain some non-negligible problems. Above all, more efforts are supposed to be devoted to the design and fabrication of wearable biosensors, which are rarely reported in the existing work. Current LC biosensors show poor processability and they are not able to be further processed into wearable devices, due to the high fluidity of the LCs. The burgeoning LC microcapsules (LC-Ms) have provided an alternative approach to realizing wearable biosensors. LC-Ms are fabricated by covering the LC droplets with polymer shells, showing satisfactory stability and processability simultaneously. By immobilizing the receptors on the polymer shells via chemical reactions, the LC-Ms with biosensing properties have been achieved. By further processing the LC-Ms using electrospinning and spin-coating, LC smart textiles can be obtained, exhibiting ideal wearability and biosensing property [97]. The development of LC-Ms makes it promising to realize the real-time detection of sweat compositions, which implies the health conditions and disease situations of the subjects.

Another key requirement is to achieve LC biosensors with gustatory simulation properties. Sour, sweet, bitter, salty, and umami are the basic tastes recognized by the taste bud cells in the human body [176]. The perceptions of these tastes can guide appetite and trigger physiological activities for absorbing nutrients and adjusting metabolism. Current studies about the gustatory simulation technologies focus on electrical sensors such as the electric cell-substrate impedance sensor [177], light addressable potentionmetric sensor [178], and multi-electrode array sensor [179], facing the problems of low detection flux and sensitivity. Considering the outstanding optical modulation property of the LCs, the optical gustatory sensors holding low LOD and high efficiency are supposed to be achieved. The main challenge of fabricating the LC gustatory sensors is the substantial diversity and redundancy of the tastant receptors. Integrating gustatory cells and LC ATP sensors together is a promising strategy to overcome the challenge. The gustatory cells in the sensors can transform the tastant signals into ATP signals, and the LC phase can further process the ATP signals into optical images, and therefore avoid the use of complex tastant receptors. In order to achieve such a sensor, the biocompatibility of the LC phase should be improved to ensure the long-term survival of the gustatory cells.

Stable sensing platforms are necessary for realizing the commercialization of LC biosensors. A variety of unstable factors can influence the sensing property of the LC biosensors, such as the immobilization amount of the receptors, the thickness of the LC cell, and the additive amount of the target solution, which are hard to be precisely controlled by human beings. Simplified fabrication processes and standardized production equipment are in urgent need to avoid these unstable factors. Moreover, the precise classification of the optical signals, as well as the accurate prediction of the sensing performance are also not artificially feasible. A tiny difference in the optical signals usually implies a distinction of the biomolecule type and concentration. Furthermore, the quantification of the biomolecules is crucial but remained unconsummated. While investigating the relationship between the biomolecular concentration and the gray level of the optical signal is an alternative solution, the task is also hard to achieve artificially. Machine learning-based strategies can eliminate the human factor and seem to be an ideal way for solving the above challenges. In recent years, efforts have been devoted by the Abbott group to develop various machine learning algorithms that can optimize the recognition accuracy and speed of the LC sensors, providing guidance for the combination of the LC biosensors and machine learning technologies [180,181]. Overall, current work has demonstrated the superiority of the LC biosensors in a variety of aspects. The sensors are of great practical value and hold promising application potential, especially after the remaining problems are solved.
